# In Melting Points We Trust: A Review on the Misguiding Characterization of Multicomponent Reactions Adducts and Intermediates

**DOI:** 10.3390/molecules27217552

**Published:** 2022-11-04

**Authors:** Brenno A. D. Neto, Pedro S. Beck, Jenny E. P. Sorto, Marcos N. Eberlin

**Affiliations:** 1 Laboratory of Medicinal and Technological Chemistry, Institute of Chemistry (IQ-UnB), Campus Universitário Darcy Ribeiro, University of Brasília, Brasília 70910-900, DF, Brazil; 2 Departamento de Química Fundamental, Universidade Federal de Pernambuco, Recife 50670-901, PE, Brazil; 3 MackMass Laboratory, PPGENM, School of Engineering, Mackenzie Presbyterian University São Paulo, São Paulo 01302-907, SP, Brazil

**Keywords:** multicomponent reactions, melting points, mechanisms, catalysis, review

## Abstract

We discuss herein the problems associated with using melting points to characterize multicomponent reactions’ (MCRs) products and intermediates. Although surprising, it is not rare to find articles in which these MCRs final adducts (or their intermediates) are characterized solely by comparing melting points with those available from other reports. A brief survey among specialized articles highlights serious and obvious problems with this practice since, for instance, cases are found in which as many as 25 quite contrasting melting points have been attributed to the very same MCR adduct. Indeed, it seems logical to assume that the inherent non-confirmatory nature of melting points could be vastly misleading as a protocol for structural confirmation, but still many publications (also in the Q1 and Q2 quartiles) insist on using it. This procedure contradicts best practices in organic synthesis, and articles fraught with limitations and misleading conclusions have been published in the MCRs field. The drawbacks inherent to this practice are indeed serious and have misguided MCRs advances. We therefore suggest some precautions aimed at avoiding future confusions.

## 1. Introduction

**Scheme 1:**
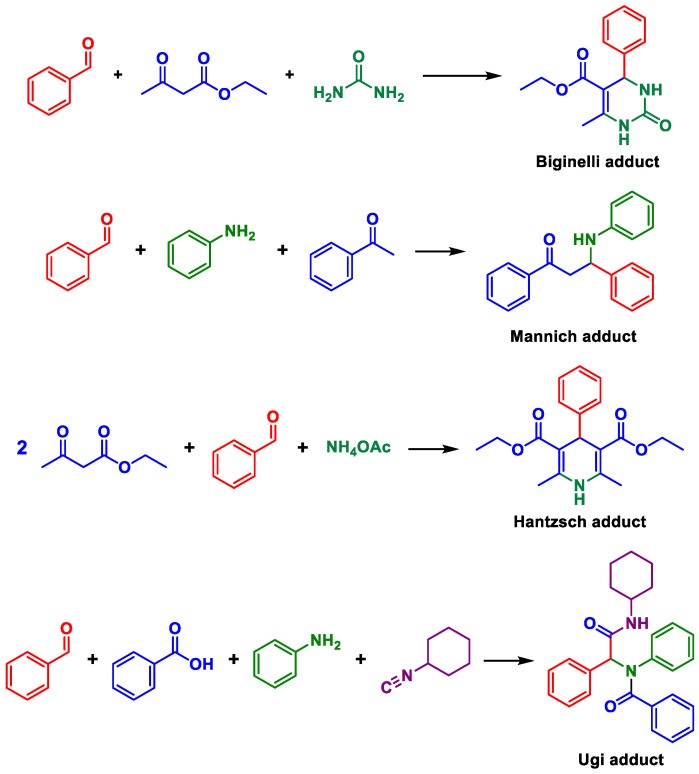
Examples of popular and widely studied multicomponent reactions.

Multicomponent reactions (MCRs) are key transformations included in the modern synthetic chemistry toolbox since they allow rapid construction of diverse and complex libraries of bioactive compounds. Recent reviews on different types and aspects of MCRs exemplifies their prominence [1,2,3,4,5,6,7,8,9]. Among the most popular and studied types are, for example, the Ugi, Passerini, Biginelli, Hantzsch, and Mannich MCRs (Scheme 1). Their mechanisms, [10] solvent effects influence, [11] catalytic approaches, [12] and general aspects [13] have also been intensively discussed and debated.

Development in MCRs is notorious, especially over the last two decades [14,15,16,17,18,19,20]. Today we have, via these multicomponent transformations, rapid and one-pot access to diverse and complex libraries of bioactive compounds and to synthetic molecules with technological potentials [21]. MCRs are also a large avenue of opportunities, such as for new catalysts, material applications, and discovery of new reactions. Their multiple-bond-forming nature is another attractive feature of MCRs [22], whereas controlling chemo-, regio- and stereoselectivities have fostered several new MCRs variants [23]. However, not all is to be praised in the MCRs field. Alongside with the MCRs blooming, as is unfortunately usual for rapidly expanding fields in science, some crucial problems have also emerged. For instance, an increasing number of MCRs articles have been retracted particularly for incorrect structural assignments and reproducibility issues, such as regarding yields and selectivities.

One major and common problem is related to poor characterization of MCRs adducts and intermediates performed solely by comparing their melting points with those of previously reported compounds. The lack of more secure structural analysis, such as that provided by mass spectrometry and nuclear magnetic resonance spectroscopy, as we will discuss, has led to misleading conclusions. The problem is more commonly noted for some popular variants, such as Biginelli, Hantzsch, and Mannich MCRs, but as we will discuss, MCRs adducts and intermediates have displayed similar spectra and melting points. Thus, mechanistic analyses and final products have drastically suffered from this bias, and many misguiding conclusions have been drawn. To give an idea of the large dimension of the problem, some MCRs products, as we have found, exhibited more than 25 contrasting reported melting points. 

In this critical overview, we highlighted that distinct mechanisms and structures for MCRs intermediates and products have been reported exclusively by the “melting point-only” characterization and we also suggest procedures to eliminate this damaging practice in MCRs studies and publications. 

## 2. “Melting Point-Only” Characterization and Its Consequences to the MCRs Arena

To didactically introduce the vital importance of the melting point (mp), we shall cite the words of Yalkowsky and Alantary: “*The melting point is by far the most commonly reported property of organic compounds. It is often among the first properties determined after a new compound is synthesized. There is probably more melting point data available for organic compounds than any other physical, chemical, or biological property*” [24].

No chemist would therefore dispute the importance of mp analysis to structural characterization, including MCRs intermediates and products. Since mps are the temperature range at which compounds melt, this easy-, inexpensive- and rapid-to-obtain physicochemical data is very useful to indicate purity and partially to characterize a structure when reliable mps are available. However, mps are not conclusive since many compounds with even very contrasting structures may display very similar mps. 

**Figure 1:**
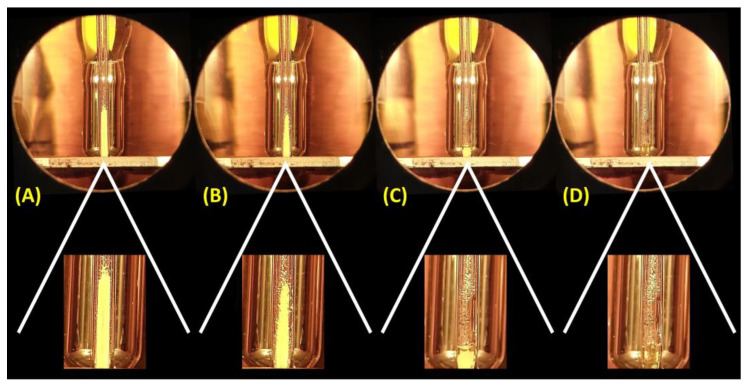
Pictures of a mp measurement of an MCR adduct using a common capillary-type melting range measuring instrument and respective expansions. From left to right: (**A**) sample before heating (1 °C min^−1^), (**B**) at the temperature of the collapse point, (**C**) the meniscus point, and (**D**) the clear point with small droplets also noted in the tube.

As Figure 1 illustrates, well-executed mp measurement should provide a narrow temperature range from the collapse point (melting starts) to the clear point (melting is completed). This range can be as wide as 2 °C of variation but is preferably as narrow as 1 °C. Slow heating is highly recommended near the melting point since the slower the heating, the better the accuracy. Unfortunately, mps with 3, 4, or even more degrees (up to 7 °C) of variation have been reported in measurements of either impure samples or with heat inputs above 1 °C min^−1^. Problems associated with heat ramps in mp measurements have been discussed elsewhere [25]. Being able to recognize the meniscus point, when both solid and liquid are in equilibrium (see Figure 1), is also crucial since it is sometimes confused with the collapse point, which occurs a few tenths of a degree lower. The mp is also sometimes reported as a single temperature, but this temperature is not technically the mp and more likely describes either the collapse, meniscus, or clear point. Typically, it refers to the meniscus point. These deviations from standard mp measurements may explain differences of a few degrees for some catalogues or specialized literatures, but it is not a plausible reason for compounds supposedly with the same structure having mps that considerably contrast. 

Again, we should always remember that mps are indeed useful, but not confirmative, and should never replace spectroscopic/spectrometric characterizations. Unfortunately, however, it is common to find reports basing structural characterization of MCRs intermediates and adducts on mere “mp-only” comparisons, thus making them prone to misleading interpretations. This is likely even a common trend. Much less susceptible to errors are mixed mp measurements, but even this simple and highly recommended procedure is rarely used. By simultaneously measuring mps of the target compound and an authentical sample in three different capillaries (one with the compound under analysis, the second with an authentical pure sample and the third with a mixture of them), a far more reliable piece of evidence for the claimed adduct are provided if an authentic sample is available for the experiment. 

The mp is also excellent for purity checking since impurities may strongly influence the result even at very low concentrations. Impurities indeed lower and broaden the mp to much greater extents due to eutectic phase [26] formation, which in turn always depresses the values of mps [27]. For example, impurities at concentrations below those typical in which spectroscopic methods reach their limits may significantly decrease mps [27], but one should always remember that mps do not replace more selective techniques, such as chromatography coupled with mass spectrometry or nuclear magnetic resonance. However, even simple mps of MCRs intermediates and adducts are often lacking. 

Since MCRs are often applied to synthesize libraries of compounds bearing several biological responses, polymorphism is also an important issue for these compounds. Polymorphism appears when compounds exist as two or more crystalline phases [28] that display different arrangements or conformations in the crystal lattice [28]. This property is crucial for mps measurements since different solid states phases and transitions associated with those phases are commonly observed in polymorphic crystals [29,30]. Thermoanalytical methods are therefore widely applied to investigate polymorphism [31,32]. Since mps measurements are typically affected by polymorphism [28], additional well-conducted XRD analysis is the least required for the proper characterization of polymorphs.

Considering that various and significantly contrasting mps of several commonly studied MCR adducts and intermediates have been reported, as we will discuss, polymorphism should indeed be considered in principle. Polymorphs may be part of this problem, particularly when contrasting colors, appearances (such as needle crystals), shapes, and other features are reported for the very same MCR adduct or intermediate synthetized under sometimes contrasting synthetic conditions, such as solvent, temperature, and catalysis. However, when similar purification processes, such as the same chromatographic column or crystallization protocols using the same solvent, are employed, polymorphs began to be less and less likely as an explanation for such contrasting mps. Although they cannot be fully discarded, it is unlikely that such differences in the reported mps for MCR adducts and intermediates, especially those widely investigated such as for the Biginelli or Hantzsch reactions, are due to polymorphism. 

We should mention here the rare exception of a landmark study that investigated the possibility of polymorphs for MCRs derivatives [33]. After the synthesis of curcuminoid–coumarin derivatives by a MCR using mechanochemical conditions, a technique prone to creating polymorphs [34,35,36], authors concluded that no polymorph was noted for the product using different solvents or for the solventless conditions. Several analyses (thermal and spectroscopic) were presented, and solvents of different polarities were tested. Thus, a precise conclusion of no polymorphism was provided. 

## 3. Examples of Contrasting Mps for MCRs Adducts and Intermediates

We will present mps for adducts and intermediates of a few selected widely studied MCRs and show that variations as high as 100 °C have been reported. In the next section, we will discuss cases in which proposed reaction intermediates have overlapping mps with the final MCR adduct. 

First, we surveyed mps for the most common Biginelli adduct formed when condensing urea, benzaldehyde, and ethyl acetoacetate to form one of the simplest DHPMs (3,4-dihydropyrimidin-2(1*H*)-one or -thione). For this DHPM, we found up to 25 contrasting mps that vary up to 20 °C (Table 1). The mps for possible reaction intermediates were also investigated since they are used as evidence for the three debatable Biginelli mechanisms [10] i.e., the iminium, the enamine, and Knoevenagel routes [10]. By these mechanisms, four intermediates may be isolated and characterized, but their formations typically occur in bimolecular reactions by mixing the two partner reagents under specific reaction conditions, from which these intermediates may be isolated and unambiguously characterized. Under the MCR condition, the isolation and characterization of intermediates are more demanding but may also be performed [37]. 

What Table 1 revealed was indeed worrying: mps of the imine intermediate (**Int B1**) were in the same range described in several studies for the Biginelli adduct. Something indeed should calls one’s attention when an intermediate is so “mp-similar” to the final product. This leads to the suspicious of misleading conclusions from this “mp-only” characterization.

Even more confusion may have been caused when characterizing intermediates **Int B2** and **Int B3** (Table 1) since their mps were also reported in comparable ranges (Table 1). These unexpected mps indeed point to the possibility of incorrect structural, and consequently mechanistic, assignments. A considerable difference is also noted for the mp of **Int B2** (Table 1) i.e., a 72 °C difference between the highest and lowest mps attributed to this molecule. The Biginelli MCR is among the most investigated MCRs [80]. Therefore, one can calculate the extent of the potential damage to the MCRs field caused by this practice of “mp-only” characterization. Although we will refrain from mentioning the whole list of articles with this issue, we found, in a brief survey of MCRs literature, many articles with “mp-only” characterization of MCRs adducts and intermediates that will be discussed in due course.

The Hantzsch reaction is also a widely studied MCR with five different debatable mechanisms [10,81,82]. Again, the “mp-only” problem (Table 2) for this MCR is clear with mps variations as broadly as 100 °C. Mechanism assignments are also in danger since **Int H2** (Table 2) has the same structure as **Int B4** (Table 1) and it may be formed in competitive reactions. An analysis similar to that performed for the Biginelli MCR is applied to the Hantzsch reaction. 

Another classical multicomponent transformation is the Mannich three-component MCR, also known as the direct Mannich. As noted for the Biginelli reaction, this multicomponent transformation (Table 3) seems also to be affected by the “mp-only” problem since its intermediates have also been reported with similar mps. Therefore, misleading conclusions are likely. 

Note that for the Mannich MCR, “mp-only” characterization should be incapable of differentiating **Int M1** and **Int M2** due to overlapping mps (Table 3). But depending on the reaction conditions, either **Int M1** or **Int M2** will form since these mechanisms may be operating competitively as commonly observed for MCRs [10].

## 4. Mechanistic Implications

The “mp-only” problem has also likely jeopardized several MCRs mechanistic studies. Isolation and characterization of intermediates is undoubtedly one of the best approaches to prove MCRs mechanisms, but it was common for us to observe reports describing “mp-only” characterizations of intermediates and final MCRs adducts. Since our goal is to call attention to this misleading “mp-only” practice and not to discredit any research group, we will refrain from listing these studies, but these publications are becoming more common, and they may be easily found.

An additional drawback appears when enantioselective MCRs are studied. The chiral induction step is typically evaluated for an intermediate of a specific mechanistic route. DFT calculations are characteristically performed to understand the chiral induction step in proposed transition states based on mechanistic suggestions. Therefore, for such MCRs with several possible reactional mechanisms, the characterization of intermediates that would allow for a more precise indication of the chiral induction step should be performed with the greatest care. 

**Scheme 2:**
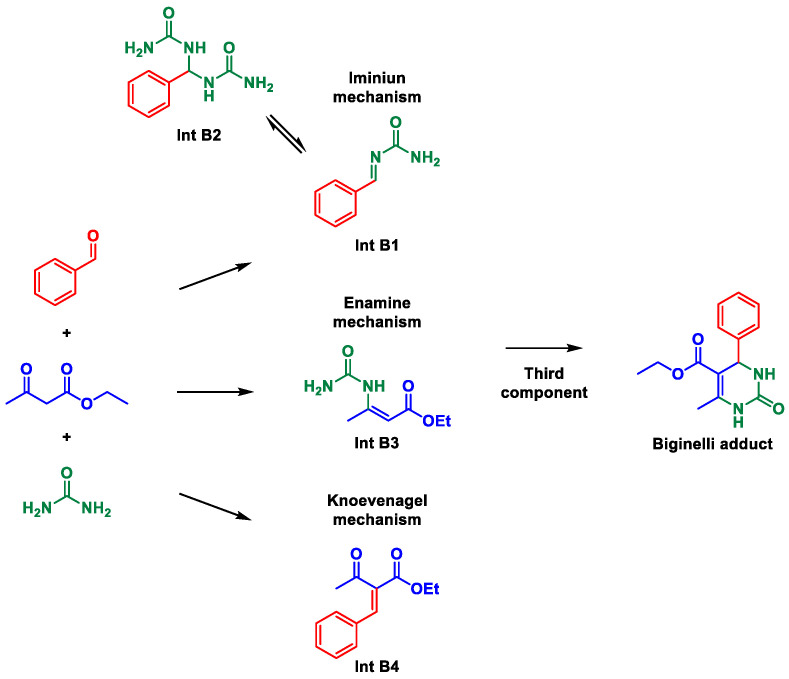
Example for the Biginelli reaction of a possible misleading conclusion reached by “mp-only” characterization of a reaction intermediate.

An initial analysis of our model Biginelli reaction exemplifies the importance of secure structural characterization (Scheme 2). If we hypothetically assume the Knoevenagel mechanism as the preferred route under a specific reaction condition, and a solid is isolated, characterized and indicated as **Int B1** (mp ≈ 203 °C, Table 1) but is actually the final Biginelli adduct—that is, the expected DHPM (mp ≈ 203 °C, Table 1), this mischaracterization would point otherwise to the iminium mechanism: an incorrect conclusion based on the misguiding “mp-only” problem. This exemplifies the damage that this practice may exert over the MCRs arena. 

Consider the same MCR again, but suppose now that a solid is isolated with an mp of ca. 158 °C. This could point to the iminium mechanism (Scheme 2) since this mp corresponds to **Int B2** (Table 1). But again, **Int B3** from the enamine mechanism (Scheme 2) has an mp of ca. 158 °C (Table 1). Again, misleading conclusions are likely from this “mp-only” characterization, hence impairing the description of the actual mechanistic route without additional characterizations.

## 5. A General View of Recent Years

Until this section, we have analyzed a few but representative and popular MCRs (i.e., Biginelli, Hantzsch, and Mannich) independent of their publication year or the Journal in which the work has been published, especially because these Journals are cited as references when convenient. During revision of this overview, reviewers fairly raised the question: *Why should we be concerned with mps if the spectral data (and spectra) of obtained organic products along with high-resolution MS or elemental analysis data are required for reporting synthetic procedures in every qualified journal?* At first, indeed, the concern with the “mp-only” problem seems unjustified; and we agreed with reviewers. However, the reality refutes this appearance. Two of us (BADN and MNE) currently work as Associate Editors in qualified journals, and we have noted similar cases in several submissions and even in published articles. Thus, this was indeed an early motivation to write about this problem.

**Figure 2:**
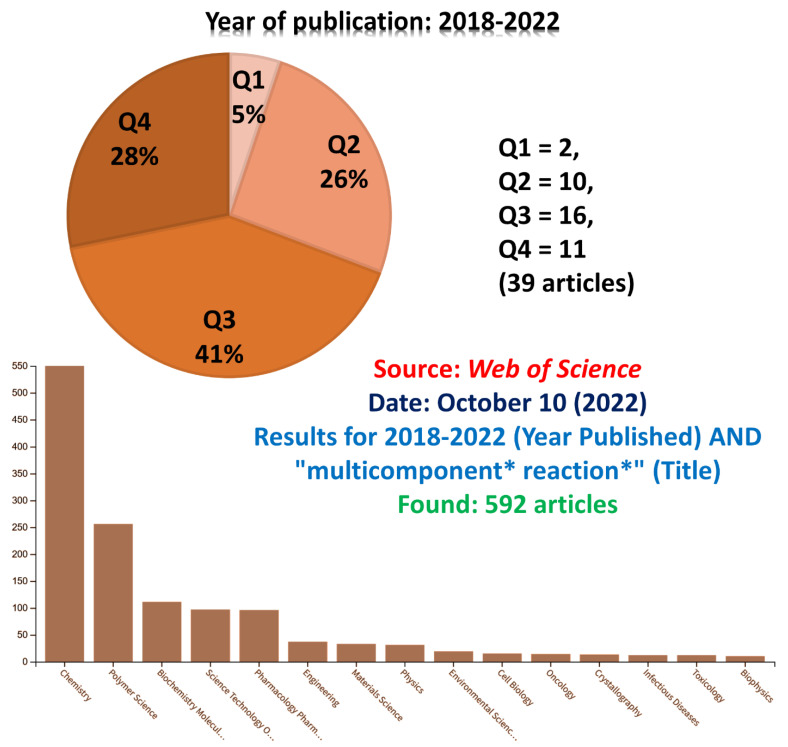
Results for 2018–2022 (year published) AND “multicomponent * reaction *” (Title). Found: 592 articles. Source: *Web of Science*.

To further highlight the “mp-only” problem, we searched for all articles indexed in *Web of Science* (Clarivate Analytics) containing the term “multicomponent * reaction *” in the Title row published in the last 5 years (2018–2022). The search returned 692 articles, and we checked them one by one. Several of them lack spectral characterization, whereas some have NMR descriptions without any available spectrum. In principle, such procedure is unacceptable but not rare at all. Since it is not our intention to discredit researchers, we included a long list of publications with the “mp-only” problem for reviewers only. However, by using the same keywords in *Web of Science*, one can easily find these articles. This initial search (Figure 2) is indeed limited and only showed the “tip of the iceberg”, since many MCRs articles lack the searched terms in their titles. Therefore, this initial screening was only useful to indicate the problem. Although it was an initial and limited analysis, the search results have already exposed some drawbacks indicated herein. Among the articles bearing the discussed problem, we noticed 5% in the Q1 (Quartile 1) and 26% in the Q2 (Quartile 2) categories; that is, ca. 30% of them have been in principle published in qualified journals. By definition, Q1 and Q2 quartiles mean a distribution of a certain category for articles published in the top 25% and 25–50%, respectively considering the Journal Impact Factor [156] from Journal Citation Reports (Clarivate Analytics). Some journals may be classified in the Q1 level considering a specific category and also be found in the Q2 considering a different category, but we considered the “Chemistry (multidisciplinary)” field since most of these articles have been published in the chemistry field (see Figure 2). The importance of the quartiles and a comparison among several different areas has been discussed elsewhere [157].

**Figure 3:**
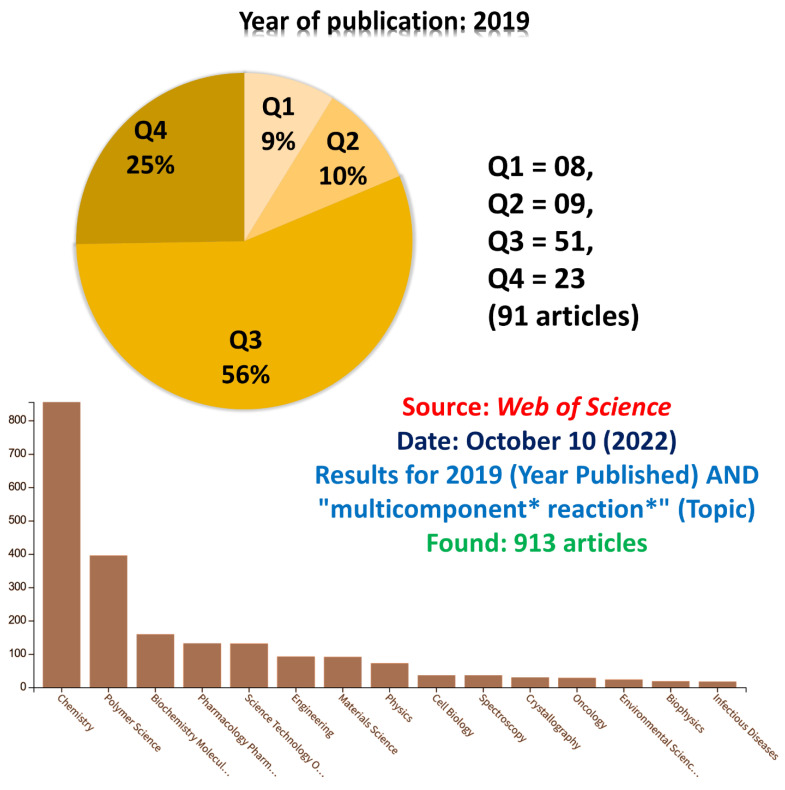
Results for 2019 (Year Published) AND “multicomponent * reaction *” (Topic). Found: 913 articles. Source: *Web of Science*.

To search more comprehensively, we randomly selected the year 2019 but now searched for the “multicomponent * reaction *” term in the Topic row instead of the Title. Nearly 1000 (one thousand) articles were returned and were checked one by one (Figure 3). As noted, for ca. 10% of all these articles, the “mp-only” issue has been noted. Among those articles with this problem, 9% are found in the Q1 and 10% in the Q2; thus, ca. 20% of them were published in good journals. Still, they display the “mp-only” problem. Even worse was to note (Figure 3) that most of these articles in which we detected the “mp-only” problem are from the chemistry field. Indeed, although in principle, chemists should be the first to recognize the importance of proper characterization of products and intermediates, they have published, and reviewers have approved, articles with misguiding “mp-only” characterization. 

## 6. Concluding Remarks

As we have shown via striking examples and have confirmed via extensive literature screening, and as noted elsewhere also in other synthetic fields, although mps are indeed useful and of vital importance for product confirmation and purity screening, “mp-only” characterization of intermediates and adducts of MCRs has likely led to incorrect structures for final products (and intermediates) and therefore to incorrect mechanistic assignments. Unfortunately, this practice is becoming more common and may jeopardize further developments in this pivotal field of synthetic chemistry. All data provided and discussed have shown that this “mp-only” problem is not at all an isolated issue related mostly to low-quality journals. Although one should expect it in very few or ideally no publications, particularly in Q1–Q2 quartiles, a direct search in *Web of Science* has indicated a substantial number of articles there suffering from the “mp-only” problem. 

We therefore suggest that authors indeed use mp for its undeniable importance and power but never replace with mps the more selective and structural revealing spectroscopic and spectrometric characterizations. If polymorphs are described, then even more extensive thermal and spectroscopic evidence of their existence should be provided. Considering also that several contrasting mps have been described for the same MCR adduct and for some intermediates, extra care should be taken when reporting mps as structural evidence, particularly for mechanistic and stereocontrolled studies. 

## Figures and Tables

**Table 1:** Reported mps (°C) for 4 common Biginelli reaction intermediates and for the MCR adduct (DHPM) typically formed in the model reaction.

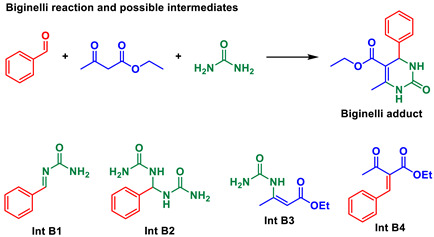					
Adduct	Reference	Int B1 (Ref.)	Int B2 (Ref.)	Int B3 (Ref.)	Int B4 (Ref.)
			225–226 ([38])		
218	[39]				
213–214	[40]				
211–212	[41]				
210–211	[42]				
210	[43]				
209–211	[44]				
209–210	[45,46]				
208–209	[47]				
207	[48]				
206–207	[49,50]				
204–207	[51]	202–205 ([41])			
204–206	[38,52,53]				
204–205	[54]				
204	[55]				
202–205	[56]				
202–204	[57]				
202–203	[58,59,60]				
201–204	[61]				
201–203	[62,63]				
201–202	[64,65]				
200–201	[66]				
200–202	[67]				
199–201	[68]				
198–200	[69,70]				
198–199	[71]				
			194 ([72])		
			178 ([73])		
			168–170 ([74])		
			157–158 ([75])	158–160 ([63])	
			154 ([76])		
					78–80 ([77])
					44–45 ([78,79])

**Table 2:** Reported mps (°C) for the Hantzsch ester derivative and some reaction intermediates of the MCR.

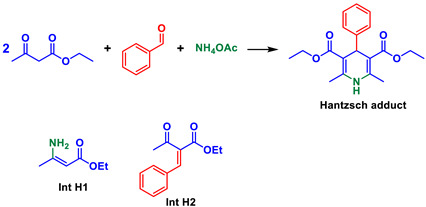			
Adduct	Reference	Int H1 (Ref.)	Int H2 (Ref.)
253	[83]		
160	[84]		
158–160	[85,86,87,88,89]		
158	[90]		
157–159	[91,92,93]		
157–158	[94,95]		
156–158	[96]		
156–157	[97]		
156	[98,99,100]		
154–155	[101]		
153–155	[102]		
153	[103]		
152–154	[104]		
150–153	[105]		
145–146	[106]		
			78–80 ([77])
			44–45 ([78,79])
		33–35 ([107])	
		32–35 ([108])	
		30–32 ([109])	

**Table 3:** Reported mps (°C) for the Mannich MCR and its proposed intermediates.

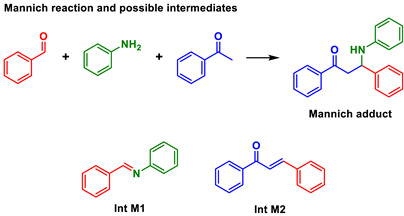			
Adduct	Reference	Int M1 (Ref.)	Int M2 (Ref.)
176–179	[110]		
174–176	[111]		
173–175	[112]		
172–174	[113,114]		
172–173	[115]		
171–172	[116]		
170–172	[117]		
170–171	[118,119]		
169–170	[120,121,122]		
168–170	[123,124]		
168–169	[125]		
167	[126]		
163–165	[127]		163–164 ([128])
161	[129]		
		140–141 ([130])	
			58 ([131])
			57 ([132])
		51–56 ([133])	56–58 ([134,135,136])
		56 ([137])	56–57 ([138,139]) 56 ([140]) 55–57 ([141,142]) 55–56 ([143]) 54–56 ([144]) 54–55 ([145]) 54 ([146])
		52 ([147])	53–54 ([148,149]) 52–53 ([150]) 52 ([151])
		51–52 ([152,153,154])	
		48–50 ([155])	
